# Some Particulars of a Fever Which Prevailed among the Crew of His Majesty's Ship Montague, at Gibraltar, in the Months of January and February, 1816

**Published:** 1816-11

**Authors:** William Shoveller

**Affiliations:** Surgeon, R. N.


*Some Particulars of a Fever which prevailed among the Crew of His Majesty's Ship Montague, at Gibraltar, in the Months of January and February,* 1816. By William Shoveller, Surgeon, R. N.

During the months of January and February 1816, there was a constant succession of cold winds and deluges of rain at Gibraltar. The crew were consequently dull and inactive; and anxious to return to their native soil, to enjoy the pleasures of the shore after a long service of seafaring life. In this period of corporeal inactivity and mental irritability, a full diet probably contributed to predispose to disease, in conjunction with the unseasonable state of the weather. A fever of considerable variety, therefore, broke out in the ship and extended to 110 of the crew. The accession of the fever, in most cases, was sudden; particularly the head-ache, which was violent, and chiefly confined to the forehead and temples. To this were added giddiness; nausea; chilliness succeeded by heat and thirst, but not very violent vascular action. Universal pains were complained of, and a great sense of debility. The tongue variable from white to brown. In a majority of the cases, the determination was evidently to the lungs, evinced by cough and increased secretion from the bronchia. In a few cases, the gastric system bore the onus of disease, determined by bilious vomiting, and in ten of these cases there was a yellow suffusion on the skin, similar to that in the endemic fevers of tropical climates. Two fatal cases only occurred; one, pneumonia typhodes; the other appeared to be a well marked case of yellow fever, but shewing at its commencement such apparently unequivocal symptoms of asthenia, that the lancet was not used. He died on the 14th day; the other on the 9th.

The treament hinged on the antiphlogistic and evacuant plan. The lancet was employed in the first hours of the attack; but we found that, excepting among the most robust and plethoric subjects, we could seldom take more than twenty, twenty-five, or thirty ounces of blood at the first operation; and that a second was inadmissible, so decided were the symptoms of debility that ensued. But the bowels were kept in constant action, with calomel, jalap, the saline cathartics, &c. with the greatest benefit. To obviate the local determinations, local remedies were used of course, and need not be specified here. That the fever was contagious, we had no reason to believe; but the yellow suffusion and bilious vomitings were somewhat remarkable at this season of the year, and during such a constitution of the atmosphere as then prevailed. The venæsection, without once exhibiting a buff coat, was invariably followed by a remission of all the violent symptoms, and its repetition only, or excess in the first instance, was productive of typhoid degeneracy. It is somewhat singular also, that subsequently on our passage home, a violent case of pneumonia, treated by active depletions, veered suddenly round to complete yellow fever, with considerable determination to the hepatic system, and was not subdued till ptyalism was induced by the submuriate of quicksilver.

Into the dispute relative to the nature and treatment of the Gibraltar fever, I do not wish to enter; but state the above facts for the consideration of both parties. The ever varying features of endemics and epidemics, determined by various, and often unseen causes, are likely to produce endless litigations amongst medical observers; and to give rise to nearly as many doctrinal and practical creeds, as there are visitations of these awful scourges! On this account, physicians should be lenient to each other, and cautious how they proclaim dogmatically this or that doctrine as alone true; this or that practice as alone successful.

WILLIAM SHOVELLER.

*October*, 15, 1816.

